# Health Survey in Gypsum Mines in India

**DOI:** 10.4103/0970-0218.58396

**Published:** 2009-10

**Authors:** Subroto S Nandi, Sarang V Dhatrak, Debasis M Chaterjee, Umesh L Dhumne

**Affiliations:** Occupational Medicine, National Institute of Miners' Health, JNARDDC Campus, Nagpur - 440 023, India

**Keywords:** Gypsum mine, miners, morbidity, pulmonary impairment

## Abstract

**Background:**

Mining is a hazardous occupation in which workers are exposed to adverse conditions. In India, gypsum mining is mainly carried out in the state of Rajasthan, which contributes about 99% of the total production.

**Objective:**

The present study was carried out in 12 different gypsum mines in Rajasthan state to determine the health status of the miners.

**Materials and Methods:**

One hundred and fifty workers engaged in mining activities were included in the study and their health status was compared with that of 83 office staff of the same mines. The health status of the employees was evaluated using a standardized medical questionnaire and pulmonary function testing.

**Statistical Analysis:**

The unpaired ‘t’ test was used to determine whether there was any significant difference between the miners and the controls and the chi-square test to compare the prevalences of various respiratory impairments in workers with that in controls; we also examined the differences between smokers and nonsmokers.

**Results:**

Our findings show that the literacy rate is low (42%) among the miners. Pulmonary restrictive impairment was significantly higher amongst smokers as compared to nonsmokers in both miners and controls. Hypertension (22.6%), diabetes (8.8%), and musculoskeletal morbidity (8%) were the common diseases in miners.

**Conclusion:**

This study shows that there is high morbidity amongst miners, thus indicating the need for regular health checkups, health education, use of personal protective devices, and engineering measures for control of the workplace environment.

## Introduction

Mining is one of the major occupations in India, employing a large workforce that is likely to grow. Mining is a hazardous occupation, with workers exposed to adverse conditions; apart from the need for hard physical labor, there is exposure to stress and environmental pollutants like dust, noise, heat, vibration, poor illumination, radiation, etc.

In India, gypsum mining is mainly carried out in the state of Rajasthan, which contributes about 99% of the total production; the remaining 1% is contributed by Jammu and Kashmir and Gujarat.(1) Gypsum is a very soft mineral composed of calcium sulfate dihydrate, with the chemical formula CaSO_4_·2H_2_O.(2) It commonly occurs as bedded deposits. It is one of the important industrial minerals in India. In addition to mineral gypsum, sea water and phosphoric acid plants are important sources of gypsum.(1) The principal uses of gypsum are in the manufacture of surgical plasters, fertilizers, pottery, cement, chemicals, and as an extender in paints.(3) There were 46 reported mines in 2003–04. The average daily labor employed in gypsum mines is 396.(4) The present study was carried out in the gypsum mines of Rajasthan to study the health status of the miners.

## Materials and Methods

The present study was carried out in 12 different mines in Rajasthan state. One hundred and fifty workers engaged in mining activities were included as the study group; they were compared with 83 office staff of the same mines who formed the control group. Consent was taken from all participants.

### Medical questionnaire

The health status of the employees was evaluated using a questionnaire modified from the standardized British Medical Research Council version.(5) Data were collected on family and personal history, work history, present and past medical conditions, symptoms and signs related each body system, etc.

### Pulmonary function test

Spirometry of the 150 miners and 80 of the 83 subjects in the control group was carried out using a Cosmed Pony Graphic 4.0 spirometer following the standard procedure.(6) Three readings were obtained for each worker and the best reading was taken for reporting and analysis. The results were interpreted as normal spirometry or obstructive, restrictive, or combined impairment. Predictive FVC was calculated using the predictive equation of Kamat *et al.* (1982).(7)

### Statistical analysis

The unpaired ‘t’ test was performed to determine whether there was any significant difference between the miners and the control group. The prevalence rates of various respiratory impairments observed in the workers were compared to that in the controls, and between smokers and nonsmokers, using the Chi-square test.

## Results

Personal information about the subjects, such as age, height, weight, body mass index (BMI), and whether habituated to smoking/tobacco chewing habit, are presented in Table 1. The age, height, weight, and BMI of the control and miner groups were comparable, no significant differences were noticed. About 10% of the miners were illiterate. The subjects exposed to mining activities are further subgrouped according to the duration of occupational exposure; this showed that the majority, both among miners and controls, had been in employment for 11–30. Pulmonary function test showed that 10% had restrictive impairment and 3.33% had obstructive impairment among the miners, whereas 9.63% had restrictive impairment and 2.40% had obstructive impairment in the control group. Pulmonary restrictive impairment was significantly higher in the smokers (as compared to nonsmokers) among the miners as well as the controls [Table 2]. There was no significant difference between smokers in the control group compared to smokers in the miner group or between nonsmokers in the control group and nonsmokers in the miner group shown in Table 3. The observed morbidity pattern is shown in Table 4. The prevalence of hypertension (systolic blood pressure > 140 and diastolic blood pressure > 90) in miners was 22.66% while it was 20.48% in the control group. Ischemic heart disease was seen in 0.66% in miners and in 2.4% in the control group. Eight percent of the miners had musculoskeletal symptoms. Diabetes (random blood sugar > 200 mg/dl) was present in 8% of the miners and 2.40% of the controls. Hyperthyroidism was seen in one miner. Asthma was present amongst 1.33% of the miners. A history of pulmonary tuberculosis was present in 1.33% of the miners and in 3.61% of the control group.

**Table 1:** Personal information, educational status, and duration of work exposure of miners and control subjects

**1) Personal information of miners and control subjects**			
Parameters	Miners (n = 150)	Control (n = 83)	*P* values
Age (yr)*	43.67 ± 7.86	44.15 ± 5.72	NS
Height (cm)*	164 ± 7.03	164.81 ± 7.07	NS
Weight (kg)*	66.28 ± 11.59	67.31 ± 10.78	NS
BMI (kg/m^2^)*	24.30 ± 3.77	24.78 ± 3.68	NS
Smokers	42 (28)	26 (31)	NS
Tobacco chewers	34 (23)	21 (25)	NS
**2) Educational status of the subjects:**			
Education	Miners (n = 150)	Control (n = 83)	
Illiterate	16 (10.66)	4 (4.81)	
Primary	47 (31.33)	2 (2.40)	
Secondary	46 (30.66)	28 (33.73)	
University	41 (27.33)	49 (59.03)	
**3) Number of subjects according to work exposure**			
Duration (yrs)	Miners (n = 150)	Control (n = 83)	
0–10	20 (13.33)	5 (6.02)	
11–20	74 (49.33)	53 (63.85)	
21–30	43 (28.66)	21 (25.30)	
> 30	13 (8.66)	4 (4.81)	

* X ± SD; figures in parentheses are in percentage; NS: nonsignificant

**Table 2:** Findings of pulmonary function test amongst smokers in miners as well as control group as compared to nonsmokers

PFT	Miners (*n* = 150)			Control (*n* = 83)		
		
	Smoker	Nonsmoker	*P* value	Smoker	Nonsmoker	*P* value
Normal	31	98		20	49	
Obstructive impairment	2	3	NS	1	1	NS
Restrictive impairment	8	7	<0.05	4	4	<0.05
Combined impairment	1	0	<0.5	0	1	NS
Not performed	0	0		1	2	

NS: Nonsignificant

**Table 3:** Findings of pulmonary function test amongst smokers of control compared to miners group as well as nonsmoker of control and miners group

PFT	Smoker			Nonsmoker		
		
	Miner	Control	*P* value	Miner	Control	*P* value
Normal	31	20	NS	98	49	NS
Obstructive impairment	2	1	NS	3	1	NS
Restrictive impairment	8	4	NS	7	4	NS
Combined impairment	1	0	NS	0	1	NS
Not performed	0	1	NS	0	2	NS

NS: Nonsignificant

**Table 4:** Morbidity pattern of subjects

Morbidity	Miners (*n* = 150)	Control (*n* = 83)
Cardiovascular system		
Hypertension	34 (22.66)	17 (20.48)
Ischemic heart disease	1 (0.66)	2 (2.40)
Musculoskeletal system		
Backache	4 (2.66)	0
Joint pain	7 (4.66)	0
Muscle cramps	1 (0.66)	0
Metabolic disorders		
Diabetes	12 (8.0)	2 (2.40)
Hyperthyroidism	1 (0.66)	0
Respiratory system		
Asthma	2 (1.33)	0
Pulmonary tuberculosis	2 (1.33)	3 (3.61)

Figures in parentheses are in percentages

## Discussion

There are very few studies on the general health status of miners. Most of the studies have focused on the prevalence of pneumoconiosis, mainly silicosis. This is probably the first study that has sought to examine the general health status of gypsum miners in India.

According to our findings, the literacy rate is poor among miners; 42% of miners were either illiterate or had been educated only up to the primary school level, as compared to 7% among the control group. Smoking was found to be more common in miners. Studies have shown that predisposing factors like smoking play an important role in aggravating lung disease in this occupational group.(8) Lung function impairment was relatively higher in miners than in controls, which could be attributable to the working conditions and the higher prevalence of the smoking habit. Pulmonary restrictive impairment was significantly higher amongst smokers in both groups. The fact that there was no significant difference between smokers in the control group and smokers in the miner group, or between nonsmokers in the control group and nonsmokers in the miner group, implies that the restrictive respiratory impairments were attributable to smoking rather than to the mining activities. Musculoskeletal symptoms were more common in the miners than in the controls, which may be attributable to the heavy physical work undertaken by the former and the exposure to machinery vibration. The prevalence of hypertension and diabetes was more among miners, which may because of the stress of the work environment.

In conclusion, we recommend that there should be regular periodic health examination and health education, and the use of personal protective equipments by the workers should be promoted. Implementing engineering measures to control exposure levels will significantly benefit the health and productivity of miners. Enforcing legal regulations, especially with regard to environmental monitoring, will ensure better working conditions. Awareness regarding prevention of health hazards in the mining industry should be created among the mine management by conducting training and education programmes.
